# Parasite fauna of the Antarctic dragonfish *Parachaenichthys charcoti* (Perciformes: Bathydraconidae) and closely related Bathydraconidae from the Antarctic Peninsula, Southern Ocean

**DOI:** 10.1186/s13071-017-2176-7

**Published:** 2017-05-12

**Authors:** Julian Münster, Judith Kochmann, Juline Grigat, Sven Klimpel, Thomas Kuhn

**Affiliations:** Goethe-University, Institute for Ecology, Evolution and Diversity; Senckenberg Biodiversity and Climate Research Centre; Senckenberg Gesellschaft für Naturforschung, Max-von-Laue-Str. 13, 60438 Frankfurt/Main, Germany

**Keywords:** Antarctica, Feeding behavior, Bathydraconinae, *Parachaenichthys charcoti*, *Gerlachea australis*, *Gymnodraco acuticeps*, *Racovitzia glacialis*, Parasites, Host specificity

## Abstract

**Background:**

As members of the Notothenioidei - the dominant fish taxon in Antarctic waters - the family Bathydraconidae includes 12 genera and 17 species. The knowledge of these species inhabiting an isolated environment is rather fragmentary, including their parasite fauna. Studies on fish hosts and their associated parasites can help gain insights into even remote ecosystems and be used to infer ecological roles in food webs; however, ecological studies on the Bathydraconidae are scarce.

**Results:**

In this study, stomach contents and parasite fauna of the Antarctic dragonfish species *Parachaenichthys charcoti* (*n* = 47 specimens) as well as of *Gerlachea australis* (*n* = 5), *Gymnodraco acuticeps* (*n* = 9) and *Racovitzia glacialis* (*n* = 6) were examined. The parasite fauna of *P. charcoti* consisted of eight genera represented by 11 species, with three of them being new host records. Overall, 24 parasite genera and 26 species were found in the sampled fish, including eleven new host records.

**Conclusion:**

Analyses revealed that the majority of the parasite species found in the different fish hosts are endemic to Antarctic waters and are characterized by a broad host range. These findings are evidence for the current lack of knowledge and the need for further parasitological studies of fish species in this unique habitat.

**Electronic supplementary material:**

The online version of this article (doi:10.1186/s13071-017-2176-7) contains supplementary material, which is available to authorized users.

## Background

Occurring in an isolated, extreme environment, the fauna inside the Antarctic Convergence is usually dominated by a high number of endemic species. These are typically embedded in food webs that consist of relatively low species numbers. This restricted species diversity is reflected in a narrow, highly specialized system of primary producers (phytoplankton, ice algae), primary consumers (zooplankton), predators (e.g. fish, whales, seals, seabirds) and detritivores [1, 2].

To date, 283 fish species are known to inhabit Antarctic waters, most of them belonging to the suborder Notothenioidei [3–5]. While economic valuable members of the family Notothenioidae where targets of a variety of studies (e.g. *Dissostichus eleginoides* [6]; *Dissostichus mawsoni* [7]), unexploited families have so far been rarely a focus of research. One example are members of the family of Antarctic dragonfishes, the Bathydraconidae. The Bathydraconidae typically occur in the demersal zone within the Antarctic Convergence, and consist of 12 genera and 17 species with a depth distribution that stretches from 5 to 1,250 m. [5].

Field observations of species occurring in the geographically isolated Antarctic are usually difficult and expensive due to a limited seasonal accessibility. In this context, parasites can help gain a better understanding of the particular fish species as they are directly linked to trophic and habitat-dependent aspects of host ecology [8–12]. Despite many studies on the parasite fauna of Antarctic fishes, most focused on the description of new species or single parasite taxa (e.g. Digenea [13]; Cestoda [14]; Nematoda [15]; Acanthocephala [16]). These studies revealed a mainly endemic parasite ensemble (e.g. [17–19]). With more than 40 known species, Digenea are the most diverse helminth parasite group [18, 20, 21]. Most of them are endemic, with benthic fish species used as intermediate host [18, 21]. In general, Antarctic fishes seem to be infected with a wide variety of parasite species, most of them with low host specificity. Nevertheless, the knowledge of the parasite fauna of members of the family Bathydraconidae remains only fragmentary [18], due to the remote sampling areas and therefore, often low sample sizes in the respective studies.

In this study, *Parachaenichthys charcoti* (Vaillant, 1906) was parasitologically examined in combination with stomach content analysis. In order to evaluate the findings, the parasite fauna of fish samples of the closely related species *Gymnodraco acuticeps* (Boulenger, 1902), *Racovitzia glacialis* (Dollo, 1900) and *Gerlachea australis* (Dollo, 1900) were assessed. The aim of this study was to extend the knowledge on the ecology of the fish species studied, their parasite fauna and parasite life-cycles and compare the findings with literature data for other members of the family Bathydraconidae.

## Methods

### Sample collection

**Fig. 1:**
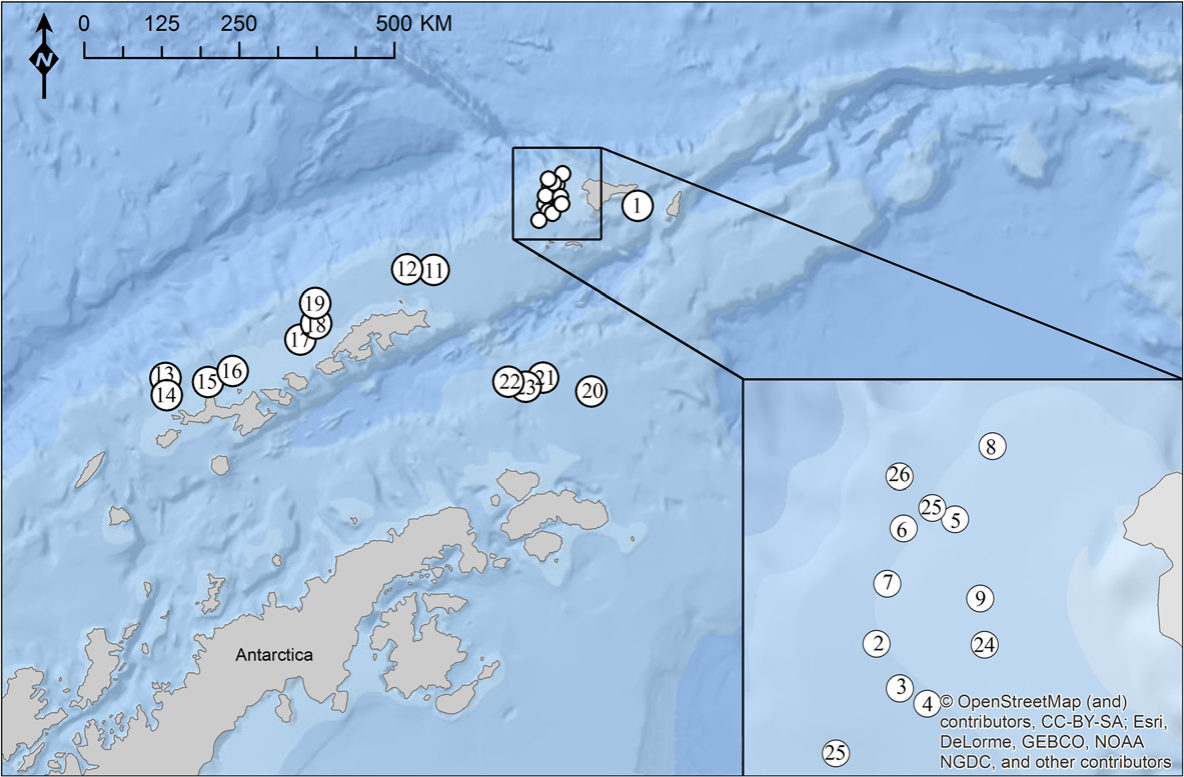
Sampling locations of the fish species studied in Antarctic waters. Coordinates of the sampling points are given in Additional file 1: Table S1

*Parachaenichthys charcoti* were caught during the research cruise ANT-XXVIII/4 in 2012 of the *RV Polarstern* in waters off the tip of Antarctic Peninsula and off South Shetland Islands (Fig. 1; Additional file 1: Table S1). The fishing was conducted with a commercially-sized 140' bottom trawl at depths between 100 and 300 m, following the standard procedure of the CCAMLR (Convention on the Conservation of Antarctic Marine Living Resources) surveys. Each haul had a towing time of 30 min with a speed of 2.6–4.4 Kn (nautical miles/h). Overall, 67 specimens of the family Bathydraconidae were caught and stored at -20 °C for examination at the Institute of Ecology, Evolution and Diversity at the Goethe University, Frankfurt/Main. Specimen identification was performed using Gon & Heemstra [22].

### Morphological and parasitological examination

As part of the morphological examination, total length (TL), preanal length (PAL), total weight (TW), and carcass weight (CW) were measured to the nearest 0.1 cm and 0.1 g. Subsequently, the body surface including skin, fins, eyes, gills as well as the nasal, buccal and branchial cavities were checked for ectoparasites. Then, the body cavity was opened and the inner organs were removed and separated. Stomach, pyloric caeca, gonads, liver and intestines were checked for endoparasites using a stereomicroscope (Olympus SZ 61, magnification × 6.7–45). For stomach content analyses, the stomach content was removed. Detected parasites were isolated and all remaining host tissues were removed carefully. For the morphological identification of the parasites, existing keys and original descriptions were used [16, 20, 23, 24]. Nematode specimens were preserved in absolute ethanol and the protocol by Münster et al. [19] was followed for molecular identification.

### Stomach content analyses

The isolated food items were separated and identified to the lowest possible taxonomic level and grouped into categories (e.g. subphylum, family, genus, species). The dry weight of the different food items as well as the empty stomach was measured and recorded to the nearest 0.001 g. For the dry weight, the food items were pat-dried with absorbent paper. Frequency of occurrence (F in %), numerical percentage of prey (N), the weight percentage of prey (W) and the index of relative importance (IRI) were calculated in accordance to Pinkaset et al. and Hyslop [25, 26].

### Data analyses

The ecological and parasitological terminology in this study followed Bush et al. [27]: prevalence (P in %) defined as the relative number of fish infected with a specific parasite; intensity (I) as the number of parasites of a particular parasite species infecting a host individual (given as a range); and mean intensity (MI) as the average intensity of a parasite species in all examined infected fish individuals. To determine the host specificity of the parasite species, the host specificity index (HSs) was calculated, using the program Specificity v1.0, following Palm & Caira [28].

In order to compare the findings of the species studied with closely related species from the family Bathydraconidae, data were collected by a broad search on Google Scholar and Web of Knowledge. Therefore, the names of the fish genera, together with the keywords “parasite”, “Digenea”, “Monogenea”, “Cestoda”, “Nematoda”, “Acanthocephala”, and “Crustacea”, were used. In addition to original publications, Klimpel et al. [29] and Oguz et al. [30] were taken into consideration. The World Register of Marine Species (www.marinespecies.org) was used for checking the validity of species names. Only unambiguous records were included.

## Results

### Host biometric and parasite infection data

In this study, 47 specimens of *Parachaenichthys charcoti*, 9 specimens of *Gymnodraco acuticeps*, 6 specimens of *Racovitzia glacialis* and 5 specimens of *Gerlachea australis* were examined for their parasite fauna and stomach content. Biometric data for the species samples are shown in Table 1. The TL for *P. charcoti* was 19.3 ± 4.7 cm (mean ± standard deviation, SD; normality test: *P* = 0.12), TW was 34.1 ± 33.9 g (normality test: *P* < 0.001) and CW was 26.8 ± 28.0 g (normality test: *P* < 0.001). Thirty-five of the 47 examined specimens of *P. charcoti* were infected with 226 metazoan parasite specimens from 8 genera and 11 species (Table 2). The most diverse and abundant group were the Nematoda (4 species), followed by Acanthocephala (3 species), Digenea (2 species), Crustacea (1 species) and Cestoda (1 species.). Nematodes were abundant with an overall prevalence of 68.1%. *Pseudoterranova decipiens* (*s.l.*) occurred in 57.5% (MI = 3.5) of the fish, followed by *Contracaecum osculatum* (*s.l*.) (P = 25.5%, MI = 3.8) and *Ascarophis nototheniae* (P = 2.1%, MI = 1). Cestodes were found in 27.6% (MI = 3.0) of the fish; all of the isolated specimens were classified as Tetraphyllidea indet. All isolated nematodes and cestodes were larval stages. Digeneans, represented by *Gonocerca phycidis* (P = 2.1%, MI = 2.0), *Lecithaster macrocotyle* (P = 2.1%, MI = 1.0), and *Lecithaster* sp. (P = 2.1%, MI = 1.0), were present in 6.4% of the examined fish. The crustacean *Eubrachiella antarctica* was only found in 2.1% (MI = 1.0). A correlation test (Spearman correlation) revealed a positive correlation for *P. charcoti* between parasite infection and TL (*r* = 0.69, *P* < 0.001) as well as TW (*r* = 0.68, *P* < 0.001). The parasite infection data for *Gymnodraco acuticeps*, *Racovitzia glacialis* and *Gerlachea australis* are listed in Tables 3, 4 and 5.

**Table 1:** Host biometric data of the fish species studied from Antarctic waters. Data are given as the mean ± standard deviation (first row), followed by the median (second row) and the range (third row)

Species	TL (cm)	TW (g)	CW (g)	LW (g)	M	F	nd
*G. acuticeps* (*n* = 9)	26.4 ± 3.50	132.06 ± 60.96	105.62 ± 50.81	3.187 ± 2.55			
	25.9	122.03	92.48	2.542	5	4	0
	20.1–32.3	48.18–264.60	37.64–219.35	1.163–9.631			
*P. charcoti* (*n* = 47)	19.3 ± 4.71	34.93 ± 33.90	26.84 ± 27.98	0.797 ± 0.88			
	16.9	19.06	15.04	0.368	16	12	19
	13.1–31.1	7.36–174.42	5.53–144.67	0.060–4.280			
*R. glacialis* (*n* = 6)	25.4 ± 5.67	84.96 ± 43.62	67.48 ± 33.70	2.083 ± 1.43			
	27.6	93.9	75.825	1.945	0	5	1
	15.1–30.0	14.14–128.49	10.98–98.40	0.294–4.140			
*G. australis* (*n* = 5)	22.2 ± 2.69	39.00 ± 18.04	32.78 ± 14.35	0.745 ± 0.52			
	22.0	34.93	30.67	0.539	4	0	1
	18.0–24.9	14.86–57.40	12.83–48.28	0.236–1.368			

*Abbreviations*: *TL* host total length, *TW* host total weight, *CW* host carcass weight, *LW* host liver weight, *M* number of male fish, *F* number of female fish, *nd* number of fish with undetermined sex

**Table 2:** Parasite fauna of *Parachaenichthys charcoti* (*n* = 47) sampled in Antarctica

Parasite	Organ	*n*	P (%)	MI	I	MA
Digenea	St, I	4	6.4	1.3	1–2	0.09
*Gonocerca phycidis* ^a^	St	2	2.1	2.0	1–2	0.04
*Lecithaster macrocotyle*	I	1	2.1	1.0	1	0.02
*Lecithaster* sp.	I	1	2.1	1.0	1	0.02
Cestoda	Bc, St, I	39	27.6	3.0	1–9	0.83
Tetraphyllidea indet.	Bc, St, I	39	27.6	3.0	1–9	0.83
Nematoda	Bc, L, P, St, I	166	68.1	5.2	1–20	3.50
*Ascarophis nototheniae*	St	1	2.1	1.0	1	0.02
*Contracaecum osculatum* (*s.l.*)	Bc, L, P, I	45	25.5	3.8	1–15	0.96
*Contracaecum radiatum*	Bc	1	2.1	1.0	1	0.02
*Contracaecum* sp.	P	1	2.1	1.0	1	0.02
*Pseudoterranova decipiens* (*s.l.*)	Bc, L, St, I	94	57.5	3.5	1–14	2.00
Nematoda indet.	Bc, L, P, St, I	24	34.0	1.5	1–4	0.51
Acanthocephala	Bc, L, P, St, I	16	21.3	1.6	1–3	0.34
*Corynosoma* cf *australe* ^a^	L	1	2.1	1.0	1	0.02
*Corynosoma bullosum*	Bc, St	4	8.5	1.0	1	0.09
*Corynosoma* sp.	P	1	2.1	1.0	1	0.02
*Metacanthocephalus dalmori*	G	4	8.5	1.0	1	0.09
Acanthocephala indet.	St, I	6	8.5	1.5	1–3	0.13
Crustacea	Bs	1	2.1	1.0	1	0.02
*Eubrachiella antarctica* ^a^	Bs	1	2.1	1.0	1	0.02

^a^New host record

*Abbreviations*: *P* (%), prevalence, *MI* mean intensity, *I* range for intensity, *MA* mean abundance, *St* stomach, *I* intestine, *Bc* body cavity, *L* liver, *P* pyloric caeca

**Table 3:** Parasite fauna of *Gymnodraco acuticeps* (*n* = 9) sampled in Antarctica

Parasite	Organ	*n*	P (%)	MI	I	MA
Digenea	I	2	11.1	2.0	2	0.22
*Neolebouria antarctica* ^a^	I	1	11.1	1.0	1	0.11
Digenea indet.	I	1	11.1	1.0	1	0.11
Cestoda	I	1	11.1	1.0	1	0.11
Tetraphyllidea indet.	I	1	11.1	1.0	1	0.11
Nematoda	Bc, L, P, St, I	115	88.9	14.4	1–66	12.78
*Contracaecum osculatum* (*s.l.*)^a^	Bc, L, P, St, I	103	88.9	12.9	1–56	11.44
*Pseudoterranova decipiens* (*s.l.*)^a^	Bc	4	11.1	4.0	4	0.44
Nematoda indet.	Bc, L, St	8	33.3	2.7	1–6	0.89
Acanthocephala	P, I	2	22.2	1.0	1	0.22
*Corynosoma bullosum* ^a^	P	1	11.1	1.0	1	0.11
Acanthocephala indet.	I	1	11.1	1.0	1	0.11

^a^New host record

*Abbreviations*: *P* (%), prevalence, *MI* mean intensity, *I* range for intensity, *MA* mean abundance, *St* stomach, *I* intestine, *Bc* body cavity, *L* liver, *P* pyloric caeca

**Table 4:** Parasite fauna of *Racovitzia glacialis* (*n* = 6) sampled in Antarctica

Parasite	Organ	*n*	P (%)	MI	I	MA
Nematoda	Bc, L, P, St, I	70	83.3	14.0	1–34	11.67
*Ascarophis nototheniae*	St	2	16.7	2.0	2	0.33
*Anisakis simplex* (*s.l.*)	St	4	16.7	4.0	4	0.67
*Contracaecum osculatum* (*s.l.*)	L, I	4	33.3	2.0	1–3	0.67
*Contracaecum radiatum* ^a^	L, P	3	33.3	1.5	1–2	0.50
*Contracaecum* sp.	L, P	42	50.0	14.0	2–24	7.00
*Pseudoterranova decipiens* (*s.l.*)^a^	L	8	33.3	4.0	1–7	1.33
Nematoda indet.	Bc, L, P, I	7	50.0	2.3	1–3	1.17

^a^New host record

*Abbreviations*: *P* (%), prevalence, *MI* mean intensity, *I* range for intensity, *MA* mean abundance, *St* stomach, *I* intestine, *Bc* body cavity, *L* liver, *P* pyloric caeca

**Table 5:** Parasite fauna of *Gerlachea australis* (*n* = 5) sampled in Antarctica

Parasite	Organ	*n*	P (%)	MI	I	MA
Digenea	I	2	20.0	2.0	2	0.40
*Neolebouria antarctica* ^a^	I	1	20.0	1.0	1	0.20
Digenea indet.	I	1	20.0	1.0	1	0.20
Nematoda	Bc, L, P, I	10	80.0	2.5	1–5	2.00
*Contracaecum osculatum* (*s.l.*)	Bc, L	3	40.0	1.5	1–2	0.60
*Contracaecum radiatum*	P	1	20.0	1.0	1	0.20
*Contracaecum* sp.	P, L	4	40.0	4.0	2	0.80
Nematoda indet.	I	2	40.0	1.0	1	0.40

^a^New host record

*Abbreviations*: *P* (%), prevalence, *MI* mean intensity, *I* range for intensity, *MA* mean abundance, *I* intestine, *Bc* body cavity, *L* liver, *P* pyloric caeca

### Stomach content analyses

The analyses of the stomach content revealed that 91.5% stomachs contained food items, mostly Crustacea (IRI = 14,091.5) and far less frequent Teleostei (IRI = 934.1) (Table 3). The Crustacea consisted predominantly of Euphausiacea (IRI = 1753.4) and Gammaridae (IRI = 126.46). Isopods (IRI = 3.4) were less common. Due to the advanced stage of digestion, identification to lower taxonomic level was not possible. Data of the other examined species are listed in Table 6.

**Table 6:** Stomach content of the examined fish species

Fish species	Food item	F (%)	N (%)	W (%)	IRI
*Gymnodraco acuticeps*	Crustacea	60.00	87.50	24.49	6719.33
	Euphausiacea	40.00	50.00	22.03	2881.35
	*Euphausia* sp.	20.00	25.00	16.09	821.78
	Crustacea indet.	20.00	37.50	2.46	799.10
	Teleostei	40.00	12.50	75.51	3520.45
*Parachaenichthys charcoti*	Crustacea	90.70	93.43	61.94	14091.53
	Euphausiacea	32.56	22.63	31.23	1753.44
	*Euphausia superba*	13.95	11.68	17.43	406.18
	Gammaridae	4.65	25.55	1.64	126.46
	Isopoda	2.33	0.73	0.72	3.37
	*Ceratoserolis* sp.	2.33	0.73	0.72	3.37
	Crustacea indet.	48.84	44.53	28.35	3559.03
	Teleostei	20.93	6.57	38.06	934.15
*Racovitzia glacialis*	Crustacea	100	100	100	20000.00
	Euphausiacea	50.00	53.33	67,42	6037.53
	*Euphausia* sp.	50.00	53.33	67,42	6037.53
	Crustacea indet.	50.00	46.67	32,58	3962.47
*Gerlachea australis*	Crustacea	100	100	100	20000.00
	Crustacea indet.	100	100	100	20000.00

*Abbreviations*: *F* frequency of occurrence, *F* numerical percentage, *W* weight percentage, *IRI* index of relative importance of the different prey groups

### Literature data analyses

Species of the family Bathydraconidae were rarely targeted in parasitological studies. Parasites of only ten members of the Bathydraconidae have been recorded in the Antarctic Convergence [30]. Overall, 36 species of metazoan parasites are known to infect specimens of the Bathydraconidae within these waters (Additional file 2: Table S2). The most abundant taxa were the Nematoda. Seven parasitic nematode species were found parasitizing all listed bathydraconid species, followed by the Digenea, found in eight species but being the most diverse group (14 species). For seven species of fish cestode parasites have been reported. Solely recorded from four fish species, Acanthocephala showed a similar to Digenea diversity (12 species). Crustacea and Hirudinea were far less abundant and diverse. The most abundant parasite species was the nematode *Ascarophis nototheniae*, occurring in five host species (*Racovitzia glacialis*, *Gymnodraco acuticeps*, *Parachaenichthys charcoti*, *P. georgianus* and *Cygnodraco mawsoni*), followed by *Contracaecum osculatum* (*s.l*.) (4 hosts), *Corynosoma bullosum* (4 hosts), *Elytrophalloides oatesi* (4 hosts) and *Neolebouria antarctica* (4 hosts). Generally, most of the known parasites show a wide fish host spectrum. For all parasite species, infecting the sampled four fish species, the host specificity index (HS_s_) showed a value between 5.5743 and 9.4542 (Table 7), indicating that all parasite species are euryxenous [28].

**Table 7:** Host specificity index for the isolated parasite species and their fish hosts. For Class, Order, Family, Genus and Species the number of taxa, parasitizing the specific host, are given

Parasite species	Class	Order	Family	Genus	Species	HS	Rank
*Neolebouria antarctica*	1	2	5	12	21	7.6701	46790515
*Elytrophalloides oatesi*	1	3	11	24	35	7.9727	93923274
*Genolinea bowersi*	1	2	6	15	31	7.6736	47163746
*Glomericirrus macrouri*	1	3	7	13	23	7.9659	92445330
*Gonocerca phycidis*	2	7	14	32	56	9.4273	26715068657
*Lecithaster macrocotyle*	1	2	4	14	19	7.6667	46421232
*Lepidapedon garrardi*	1	1	4	15	30	6.0544	1133679
*Lepocreadium trullaforme*	1	1	2	2	3	5.5743	375252
*Macvicaria georgiana*	1	1	3	9	22	5.8782	755487
*Otodistomum cestoides*	2	4	4	4	5	9.4048	2539978652
*Anisakis simplex* (*s.l.*)	2	11	17	26	39	9.4542	2846169314
*Ascarophis nototheniae*	1	3	6	12	16	7.9641	92075070
*Caudotestis glacialis*	1	1	2	2	2	5.5743	375251
*Contracaecum osculatum* (*s.l.*)	1	3	9	22	27	7.9693	93189730
*Contracaecum radiatum*	1	3	5	13	18	7.9624	91705800
*Pseudoterranova decipiens* (*s.l.*)	1	5	12	27	45	8.2624	183000359
*Aspersentis megarhynchus*	1	2	4	8	11	7.6666	46415287
*Corynosoma arctocephali*	1	2	4	10	16	7.6666	46417275
*Corynosoma australe*	1	2	3	3	3	7.6631	46038051
*Corynosoma bullosum*	1	3	10	21	30	7.9710	93555040
*Corynosoma hamanni*	1	1	3	11	23	5.8793	757469
*Corynosoma pseudohamanni*	1	1	4	13	25	6.0537	1131701
*Corynosoma shackletoni*	1	1	3	3	6	5.8747	749504
*Hypoechinorhynchus magellanicus*	1	1	3	4	5	5.8753	750500
*Metacanthocephalus dalmori*	1	2	5	11	17	7.6701	46789522
*Metacanthocephalus johnstoni*	1	1	3	5	10	5.8759	751496
*Eubrachiella antarctica*	1	1	4	4	5	6.0503	1122753

*Abbreviation*: *HS* host specificity index

## Discussion

The parasitological examination of *Parachaenichthys charcoti* revealed, compared to other members of the Nototheniodei [29], a medium diverse parasite fauna. In addition to the 19 known parasite species infecting *P. charcoti* [30], three new host records were detected within this study (Table 2). The parasite fauna of *P. charcoti* was composed of Digenea (2 species), Nematoda (4 species), Cestoda (1 species), Acanthocephala (3 species) and Crustacea (1 species). Nematoda, the most abundant group, was dominated by *Pseudoterranova decipiens* (*s.l.*). Like most nematodes within the Antarctic Convergence, *P. decipiens* (*s.l*.) shows a generalist host range for fish [31]. Its distribution in Antarctic waters is linked to the distribution and population sizes of Pinnipedia, which are very abundant final hosts and consequently maintain a constant (high) level of nematodes within the Antarctic convergence [32, 33]. Nematode specimens belonging to the complex of sibling species *Contracaecum osculatum* (*s.l.*) were the second most abundant group. Like *Pseudoterranova decipiens* (*s.l.*), *C. osculatum* (*s.l.*) uses mainly Pinnipedia as final hosts and shows a benthic life-cycle [34]. The free-living larval stages of *Contracaecum radiatum* on the other hand, that are able to stay in the water column, usually integrate pelagic hosts in their life-cycle [35]. However, *C. radiatum* (P = 2.1%) was only found once in the sampled specimens of *P. charcoti*, whereas the high infection numbers of nematodes with a benthic life-cycle, i.e. *C. osculatum* (P = 25.1%) and *P. decipiens* E (P = 57.7%), indicate demersal life-cycle for *P. charcoti*, which corresponds with former literature findings [36, 37].

In terms of diversity, Nematoda were followed by the phylum Acanthocephala. Of the three identified species, two belonged to the genus *Corynosoma*, *C. australe* and *C. bullosum. Corynosoma australe* uses marine mammals (e.g. *Hydrurga leptonyx*) as final hosts. So far, an intermediate fish host was not known from Antarctic waters, leading to the assumption that the life-cycle usually takes place outside of the Antarctic Convergence [16]; therefore, it is listed in the results as *C.* cf. *australe*. Like *C. australe*, *C. bullosum* includes pinnipeds (e.g. *Mirounga leonina*) as final host [16]. Both *Corynosoma* spp. are distributed circumpolar in Antarctic waters and beyond [16].

Interestingly, only four specimens of the usually most diverse metazoan parasite group in Antarctic waters, Digenea [18], were detected in the fish sample of *P. charcoti*. Of those three could be identified to species level within this study (*Gonocerca phycidis*: 2 specimens, *Lecithaster macrocotyle*: 1, *Lecithaster* sp.: 1). *Gonocerca phycidis* and *L. macrocotyle* are both linked to the benthic host communities in fjord and continental shelf regions within the Antarctic, with typically high infection numbers in larger predatory fish (e.g. *Notothenia rossii*) [16]. As *P. charcoti* is a rather small predatory fish, preying primarily on Crustacea with mostly very low prevalences [38], infection numbers were low (Table 2).

Overall, together with the closely related Bathydraconidae, *Gerlachea australis*, *Gymnodraco acuticeps* and *Racovitzia glacialis*, parasite infection patterns revealed the highest diversity for Nematoda in all four examined species (Tables 2, 3, 4 and 5). This pattern is different from the parasite diversity in Antarctica, where digeneans are usually known to be the most diverse parasite group, predominantly using teleosts as definitive hosts [18]. While the nematode fauna is relatively uniform, with *Contracaecum osculatum* (*s.l.*) and *Pseudoterranova decipiens* E occurring in all species studied, the composition of the digenean fauna varied between species, especially when compared with literature findings (e.g. [13, 20, 39]. For example, *Neolebouria antarctica*, a typical representative in demersal fish species from shelf and fjord systems [20], was also isolated from *G. australis*, *G. acuticeps*, and *P. charcoti*, with relatively high abundance. However, this parasite was absent from *R. glacialis*, which might be explained by the very low sample size.

The parasite diversity of the fish sampled (*Gerlachea australis*: 4 parasite species; *Gymnodraco acuticeps*: 9 spp; *Parachaenichthys charcoti*: 23 spp.; *Racovitzia glacialis*: 11 spp.) can be considered as low to medium when compared to other fish species inhabiting the same waters, e.g. *Dissostichus elegionoides* (Nototheniidae): 47 parasite species [29]; *Macrourus whitsoni* (Macrouridae): 25 spp. [19]; *Muraneonlepis marmorata* (Muraenolepididae): 29 spp. [40]; *Notothenia coriiceps* (Nototheniidae): 37 spp. [41]). One reason might be the position of these different fish species in the food web, with larger predators (e.g. *Dissostichus* spp.), often being heavily and more diversely parasitized, than smaller species (e.g. Bathydraconidae), feeding mostly on small crustaceans (this study). The same applies to infection patterns within one species; size-dependent differences in parasite infection rates as well as parasite fauna composition can be observed [11, 42]. However, another reason is that the known parasite fauna is most often directly linked to sampling effort [19]; therefore a more diverse parasite fauna is common for intensively studied fish species such as many of the economically important fishes (e.g. *Gadus morhua* from the North Atlantic, as one of the most intensive studied marine species with more than 130 known parasite species) [11, 43]. On the scale of individual studies, sample size has a similar effect, which probably explains the relatively low number of parasites found in the samples *G. australis*, *G. acuticeps* and *R. glacialis* compared to *P. charcoti*, although their position in the food web is similar. Overall, 24 parasite genera and 26 species were found in the sampled fish, including eleven new host records (*P. charcoti*: 3 new host records; *G. australis*: 1; *R. glacialis*: 3; G*. acuticeps*: 4 (Tables 2, 3, 4 and 5). The majority of these parasite species are endemic to Antarctic waters; nevertheless exceptions such as the cosmopolitan *Gonocerca phycidis* can occur [13, 20]. Although endemic to the region, all of the species that were found to infect the four fish species in this study, are euryxenous, thus, they have a wide host spectrum (e.g. *Gonocerca phycidis*, *Anisakis simplex* (*s.l.*), *Contracaecum radiatum, Corynosoma bullosum*) [6, 16, 17, 29, 44]. Only *Stenakron glacialis* has a narrow known host range [13, 20, 45]. According to the results of the literature data analyses, this pattern of a mostly euryxenous host spectrum holds true for the majority of parasites infecting species of Bathydraconidae in Antarctic waters.

Parasite host specificity can have various forms and way of developments [46]. One way is the coevolution between the parasite and its host. A high host specificity is often caused by a close coevolution between the host and the parasite, i.e. one parasite taxon is associated to one host taxon. On the other hand, a broad host range often originates from a lack of coevolution and multiple host switches [47]. Therefore, species belonging to a host group with a variety of different genera and species, often exhibit a larger parasite diversity, while host species with only few related species tend to show a poorer parasite fauna. *Macrourus whitsoni*, a member of the family Macrouridae, with only a single related species, *M. caml*, within the Antarctic Convergence, shows a very host-specific parasite fauna [19]. In contrast, the 17 species of Bathydraconidae are members of the Notothenioidei, the most dominant component of the recent Antarctic fish fauna [3]. This group is suspected to have gone through a strong diversification [48]. The pronounced diversification, as well as the co-occurrence of several closely related species may have favored host switches of the associated parasites and therefore caused the wide host range of the latter.

## Conclusion

Eleven new host records were found in this study of parasites of four different species of the Bathydraconidae. All parasite species found can be characterized by a broad host range. The high number of new host records highlights the need for further work in the Antarctic Convergence in order to better understand this unique ecosystem and the food web structures within it.

## Additional files

Additional file 1: Table S1. Catch data of the examined species from Antarctic waters. *Abbreviations*: G.a, *Gerlachea australis*; G.ac, *Gymnodraco acuticeps*; P.c, *Parachaenichthys charcoti*, R.g, *Racovitzia glacialis*. (DOCX 19 kb)

Additional file 2: Table S2. Parasite taxa of bathydraconid species, based on literature data and own studies. Species occurring outside of the Antarctic Convergence (e.g. South Georgia Island) are included. Records marked with an *asterisk* (*) were taken from Oguz et al. [30]. *Abbreviations*: D, Digenea; C, Cestoda; N, Nematoda; A, Acanthocephala; Cr, Crustacea; H, Hirudinea; P%, prevalence; MI, mean intensity; I, intensity range. (DOCX 48 kb)

## References

[1] El-Sayed SZ (1988). Productivity of the Southern Ocean: a closer look. Comp Biochem Physiol B Comp Biochem.

[2] Griffiths HJ (2010). Antarctic marine biodiversity - what do we know about the distribution of life in the Southern Ocean?. PLoS ONE.

[3] Eastman JT (1991). Evolution and diversification of Antarctic notothenioid fishes. Am Zool.

[4] Eastman JT, Clarke A, Di Prisco G, Pisano E, Clarke A (1998). A comparison of adaptive radiations of Antarctic fish with those of non-Antarctic fish. Fishes of Antarctica.

[5] Froese R, Pauly D. FishBase. World Wide Web Electron Publ. 2016. www.fishbase.org.

[6] Brickle P, MacKenzie K, Pike A (2005). Parasites of the Patagonian toothfish, *Dissostichus eleginoides* Smitt 1898, in different parts of the Subantarctic. Polar Biol.

[7] Gordeev II, Sokolov SG. Parasites of the Antarctic toothfish (*Dissostichus mawsoni* Norman, 1937) (Perciformes, Nototheniidae) in the Pacific sector of the Antarctic. Polar Res. 2016;35.

[8] Platt NE (1976). Codworm - a possible biological indicator of the degree of mixing of Greenland and Iceland cod stocks. J Cons.

[9] Williams HH, MacKenzie K, McCarthy AM (1992). Parasites as biological indicators of the population biology, migrations, diet, and phylogenetics of fish. Rev Fish Biol Fish.

[10] Oliva ME, González M, Acuña E (2004). Metazoan parasite fauna as a biological tag for the habitat of the flounder *Hippoglossina macrops* from northern Chile, in a depth gradient. J Parasitol.

[11] Münster J, Klimpel S, Fock HO, MacKenzie K, Kuhn T (2015). Parasites as biological tags to track an ontogenetic shift in the feeding behaviour of *Gadus morhua* off West and East Greenland. Parasitol Res.

[12] MacKenzie K, Hemmingsen W (2015). Parasites as biological tags in marine fisheries research: European Atlantic waters. Parasitology.

[13] Zdzitowiecki K (1991). Occurrence of digeneans in open sea fishes off the South Shetland Islands and South Georgia, and a list of fish digeneans in the Antarctic. Pol Polar Res.

[14] Rocka A, Zdzitowiecki K (1998). Cestodes in fishes of the Weddell Sea. Acta Parasitol.

[15] Palm H, Andersen K, Klöser H, Plötz J (1994). Occurrence of *Pseudoterranova decipiens* (Nematoda) in fish from the southeastern Weddell Sea (Antarctic). Polar Biol.

[16] Zdzitowiecki K, Wägele J-W, Sieg J (1991). Antarctic Acanthocephala. Synopsis of the Antarctic benthos.

[17] Walter T, Palm H, Piepiorka S, Rückert S (2002). Parasites of the Antarctic rattail *Macrourus whitsoni* (Regan, 1913) (Macrouridae, Gadiformes). Polar Biol.

[18] Rocka A (2006). Helminths of Antarctic fishes: Life cycle biology, specificity and geographical distribution. Acta Parasitol.

[19] Münster J, Kochmann J, Klimpel S, Klapper R, Kuhn T (2016). Parasite fauna of Antarctic *Macrourus whitsoni* (Gadiformes: Macrouridae) in comparison with closely related macrourids. Parasit Vectors.

[20] Zdzitowiecki K (1997). Antarctic Digenea, parasites of fishes.

[21] Zdzitowiecki K, Di Prisco G, Pisano E, Clarke A (1998). Diversity of Digenea, parasites of fishes in various areas of the Antarctic. Fishes of Antarctica.

[22] Gon O, Heemstra PC (1990). Fishes of the Southern Ocean.

[23] Byrd MA (1963). Helminth parasites of Antarctic vertebrates. Part I. Digenetic trematodes of marine fishes. Proc Helminthol Soc Wash.

[24] Laskowski Z, Jeżewski W, Zdzitowiecki K (2010). New data on the occurrence of Acanthocephala in Antarctic Amphipoda. Acta Parasitol.

[25] Pinkas L, Oliphant MS, Iverson ILK (1971). Food habits study. Fish Bull.

[26] Hyslop EJ (1980). Stomach contents analysis - a review of methods and their application. J Fish Biol.

[27] Bush AO, Lafferty KD, Lotz JM, Shostak AW (1997). Parasitology meets ecology on its own terms: Margolis et al. revisited. J Parasitol.

[28] Palm HW, Caira JN (2008). Host specificity of adult versus larval cestodes of the elasmobranch tapeworm order Trypanorhyncha. Int J Parasitol.

[29] Klimpel S, Busch MW, Kellermanns E, Kleinertz S, Palm HW (2009). Metazoan deep sea fish parasites.

[30] Oguz MC, Tepe Y, Belk MC, Heckmann RA, Aslan B, Gurgen M (2015). Metazoan parasites of Antarctic fishes. Turk J Parasitol.

[31] Rokicki J, Rodjuk G, Zdzitowiecki K, Laskowski Z (2009). Larval ascaridoid nematodes (Anisakidae) in fish from the South Shetland Islands (Southern Ocean). Pol Polar Res.

[32] Des Clers S, Andersen K (1995). Sealworm (*Pseudoterranova decipiens*) transmission to fish trawled from Hvaler, Oslofjord, Norway. J Fish Biol.

[33] Palm HW (1999). Ecology of *Pseudoterranova decipiens* (Krabbe, 1878) (Nematoda: Anisakidae) from Antarctic waters. Parasitol Res.

[34] Bullini L, Nascetti G, Paggi L, Orecchia P, Mattiucci S, Berland B (1986). Genetic variation of ascaridoid worms with different life cycles. Evolution.

[35] Klöser H, Plötz J, Palm H, Bartsch A, Hubold G (1992). Adjustment of anisakid nematode life cycles to the high Antarctic food web as shown by *Contracaecum radiatum* and *C. osculatum* in the Weddell Sea. Antarct Sci.

[36] Kock K-H (1992). Antarctic fish and fisheries.

[37] La Mesa M, Caputo V, Eastman JT (2007). Gametogenesis in the dragonfishes *Akarotaxis nudiceps* and *Bathydraco marri* (Pisces, Notothenioidei: Bathydraconidae) from the Ross Sea. Antarct Sci.

[38] Busch MW, Kuhn T, Münster J, Klimpel S, Mehlhorn H (2012). Marine crustaceans as potential hosts and vectors for metazoan parasites. Arthropods as vectors of emerging diseases.

[39] Laskowski Z, Rocka A, Zdzitowiecki K, Ghigliotti L, Pisano E (2005). New data on the occurrence of internal parasitic worms in the *Gymnodraco acuticeps* and *Cygnodraco mawsoni* (Bathydraconidae) fish in the Ross Sea. Antarctica Pol Polar Res.

[40] Gordeev II, Sokolov SG. Helminths and the feeding habits of the marbled moray cod *Muraenolepis marmorata* Günther, 1880 (Gadiformes, Muraenolepididae) in the Ross Sea (Southern Ocean). Polar Biol. 2016;1–8.

[41] Palm HW, Reimann N, Spindler M, Plötz J (1998). The role of the rock cod *Notothenia coriiceps* Richardson, 1844 in the life-cycle of Antarctic parasites. Polar Biol.

[42] Poulin R (2000). Variation in the intraspecific relationship between fish length and intensity of parasitic infection: biological and statistical causes. J Fish Biol.

[43] Hemmingsen W, MacKenzie K (2001). The parasite fauna of the Atlantic cod, *Gadus morhua* L. Adv Mar Biol.

[44] Palm HW, Klimpel S, Walter T (2007). Demersal fish parasite fauna around the South Shetland Islands: high species richness and low host specificity in deep Antarctic waters. Polar Biol.

[45] Zdzitowiecki K, Ozouf-Costaz C (2013). Contribution to the knowledge of the parasitic fauna of fish off Adelie Land, Antarctica. Pol Polar Res.

[46] Hoberg EP, Brooks DR (2008). A macroevolutionary mosaic: episodic host-switching, geographical colonization and diversification in complex host-parasite systems. J Biogeogr.

[47] Barker SC (1991). Evolution of host-parasite associations among species of lice and rock-wallabies: Coevolution?. Int J Parasitol.

[48] Derome N, Chen W-J, Dettaı A, Bonillo C, Lecointre G (2002). Phylogeny of Antarctic dragonfishes (Bathydraconidae, Notothenioidei, Teleostei) and related families based on their anatomy and two mitochondrial genes. Mol Phylogenet Evol.

