# Supplementary material for: Parasite fauna of the Antarctic dragonfish Parachaenichthys charcoti (Perciformes: Bathydraconidae) and closely related Bathydraconidae from the Antarctic Peninsula, Southern Ocean
Source: Parasit Vectors. 2017 May 12;10:235. doi: 10.1186/s13071-017-2176-7 (PMC5427613; doi:10.1186/s13071-017-2176-7)
Supplement: Supplementary file 1 — Catch data of the examined species from Antarctic waters. Abbreviations: G.a, Gerlachea australis; G.ac, Gymnodraco acuticeps; P.c, Parachaenichthys charcoti, R.g, Racovitzia glacialis. (DOCX 19 kb) [file 13071_2017_2176_MOESM1_ESM.docx]

**Additional file 1: Table S1.** Catch data of the examined species from Antarctic waters. *Abbreviations*: G.a, *Gerlachea australis*; G.ac, *Gymnodraco acuticeps*; P.c, *Parachaenichthys charcoti*, R.g, *Racovitzia glacialis.*

| **Nr.** | **Station** | **Date** | **Pos Lat** | **Pos Lon** | **Time**  **[min]** | **Depth**  **[m]** | ***G.a*** | ***G.ac*** | ***P.c*** | ***R.g*** | **(n)** |
| --- | --- | --- | --- | --- | --- | --- | --- | --- | --- | --- | --- |
| 1 | PS79/188  - Set  - Haul | 18.03.2012  18.03.2012 | 61° 11,22' S  61° 9,86' S | 54° 35,30' W  54° 32,99' W | 30 | 277.5  355.9 | 2 |  | 2 | 3 | 7 |
| 2 | PS79/202  - Set  - Haul | 20.03.2012  20.03.2012 | 61° 10,50' S  61° 11,29' S | 55° 55,65' W  55° 51,94' W | 30 | 124.9  121.1 |  |  | 1 |  | 1 |
| 3 | PS79/203  - Set  - Haul | 20.03.2012  20.03.2012 | 61° 12,98' S  61° 14,65' S | 55° 52,64' W  55° 54,61' W | 30 | 136.7  158.0 |  |  | 1 |  | 1 |
| 4 | PS79/204  - Set  - Haul | 20.03.2012  20.03.2012 | 61° 14,35' S  61° 16,06' S | 55° 48,88' W  55° 47,31' W | 30 | 131.8  146.9 |  |  | 1 |  | 1 |
| 5 | PS79/214  - Set  - Haul | 22.03.2012  22.03.2012 | 61° 2,58' S  61° 0,70' S | 55° 45,51' W  55° 45,00' W | 30 | 111.8  147.8 |  |  | 5 |  | 5 |
| 6 | PS79/221  - Set  - Haul | 23.03.2012  23.03.2012 | 61° 3,33' S  61° 1,51' S | 55° 51,93' W  55° 51,20' W | 30 | 152.1  139.8 |  |  | 1 |  | 1 |
| 7 | PS79/222  - Set  - Haul | 23.03.2012  23.03.2012 | 61° 7,03' S  61° 5,29' S | 55° 54,43' W  55° 52,84' W | 30 | 126.7  127.9 |  |  | 1 |  | 1 |
| 8 | PS79/229  - Set  - Haul | 24.03.2012  24.03.2012 | 60° 57,82' S  60° 59,33' S | 55° 40,59' W  55° 42,32' W | 30 | 88.8  97.2 |  |  | 1 |  | 1 |
| 9 | PS79/230  - Set  - Haul | 24.03.2012  24.03.2012 | 61° 7,59' S  61° 9,02' S | 55° 42,35' W  55° 40,55' W | 30 | 64.0  82.7 |  |  | 5 |  | 5 |
| 10 | PS79/234  - Set  - Haul | 25.03.2012  25.03.2012 | 61° 17,42' S  61° 17,33' S | 56° 1,25' W  56° 5,06' W | 30 | 279.7  306.1 |  |  | 1 |  | 1 |
| 11 | PS79/243  - Set  - Haul | 27.03.2012  27.03.2012 | 61° 38,21' S  61° 38,15' S | 57° 32,72' W  57° 34,09' W | 30 | 425.4  431.6 | 1 |  |  |  | 1 |
| 12 | PS79/244  - Set  - Haul | 27.03.2012  27.03.2012 | 61° 38,86' S  61° 38,80' S | 57° 47,52' W  57° 51,31' W | 30 | 322.2  334.0 |  |  | 1 |  | 1 |
| 13 | PS79/247  - Set  - Haul | 28.03.2012  28.03.2012 | 62° 22,19' S  62° 23,72' S | 61° 25,78' W  61° 24,45' W | 30 | 325.4  353.3 | 1 |  |  |  | 1 |
| 14 | PS79/248  - Set  - Haul | 28.03.2012  28.03.2012 | 62° 29,18' S  62° 28,15' S | 61° 24,57' W  61° 21,92' W | 30 | 120.3  141.3 |  |  | 2 |  | 2 |
| 15 | PS79/252  - Set  - Haul | 29.03.2012  29.03.2012 | 62° 23,80' S  62° 24,28' S | 60° 48,81' W  60° 44,91' W | 30 | 87.7  90.7 |  |  | 1 |  | 1 |
| 16 | PS79/253  - Set  - Haul | 29.03.2012  29.03.2012 | 62° 19,44' S  62° 20,28' S | 60° 27,39' W  60° 31,31' W | 30 | 110.9  127.8 |  | 1 | 3 |  | 4 |
| 17 | PS79/258  - Set  - Haul | 30.03.2012  30.03.2012 | 62° 6,64' S  62° 5,29' S | 59° 28,60' W  59° 31,12' W | 30 | 95.0  105.1 |  |  | 8 |  | 8 |
| 18 | PS79/259  - Set  - Haul | 30.03.2012  30.03.2012 | 61° 59,99' S  61° 59,97' S | 59° 14,73' W  59° 11,29' W | 30 | 129.1  142.8 |  |  | 4 |  | 4 |
| 19 | PS79/260  - Set  - Haul | 30.03.2012  30.03.2012 | 61° 51,74' S  61° 50,50' S | 59° 15,47' W  59° 12,16' W | 30 | 259.5  269.1 |  |  | 2 |  | 2 |
| 20 | PS79/269  - Set  - Haul | 01.04.2012  01.04.2012 | 62° 27,59' S  62° 26,14' S | 55° 15,62' W  55° 16,16' W | 30 | 227.3  254.8 |  | 1 |  |  | 1 |
| 21 | PS79/273  - Set  - Haul | 02.04.2012  02.04.2012 | 62° 22,04' S  62° 23,90' S | 55° 57,65' W  55° 58,36' W | 30 | 336.6  348.5 |  | 1 |  |  | 1 |
| 22 | PS79/275  - Set  - Haul | 02.04.2012  02.04.2012 | 62° 25,36' S  62° 23,83' S | 56° 13,61' W  56° 11,34' W | 30 | 425.3  450.1 |  | 4 |  | 3 | 7 |
| 23 | PS79/278  - Set  - Haul | 03.04.2012  03.04.2012 | 62° 25,80' S  62° 26,34' S | 56° 12,58' W  56° 16,46' W | 30 | 425.9  422.6 | 1 | 2 |  |  | 3 |
| 24 | PS79/282  - Set  - Haul | 04.04.2012  04.04.2012 | 61° 10,54' S  61° 12,24' S | 55° 41,61' W  55° 41,59' W | 30 | 88.5  96.1 |  |  | 4 |  | 4 |
| 25 | PS79/283  - Set  - Haul | 04.04.2012  04.04.2012 | 61° 1,75' S  61° 2,64' S | 55° 48,50' W  55° 51,58' W | 30 | 122.8  150.8 |  |  | 1 |  | 1 |
| 26 | PS79/286  - Set  - Haul | 04.04.2012  04.04.2012 | 61° 0,16' S  61° 1,65' S | 55° 53,06' W  55° 54,14' W | 30 | 165.8  213.3 |  |  | 2 |  | 2 |
