# Supplementary material for: Parasite fauna of the Antarctic dragonfish Parachaenichthys charcoti (Perciformes: Bathydraconidae) and closely related Bathydraconidae from the Antarctic Peninsula, Southern Ocean
Source: Parasit Vectors. 2017 May 12;10:235. doi: 10.1186/s13071-017-2176-7 (PMC5427613; doi:10.1186/s13071-017-2176-7)
Supplement: Supplementary file 2 — Parasite taxa of bathydraconid species, based on literature data and own studies. Species occurring outside of the Antarctic Convergence (e.g. South Georgia Island) are included. Records marked with an asterisk (*) were taken from Oguz et al. [30]. Abbreviations: D, Digenea; C, Cestoda; N, Nematoda; A, Acanthocephala; Cr, Crustacea; H, Hirudinea; P%, prevalence; MI, mean intensity; I, intensity range. (DOCX 48 kb) [file 13071_2017_2176_MOESM2_ESM.docx]

**Additional file 2: Table S2.** Parasite taxa of bathydraconid species, based on literature data and own studies. Species occurring outside of the Antarctic Convergence (e.g. South Georgia Island) are included. Records marked with an *asterisk* (*) were taken from Oguz et al. [30]. *Abbreviations*: D, Digenea; C, Cestoda; N, Nematoda; A, Acanthocephala; Cr, Crustacea; H, Hierudinea; P%, prevalence; MI, mean intensity; I, intensity range.

| **Hosts** | **Depth [m]** | **Taxa** | **Parasiten** | **Region** | **P%** | **mI (I)** | **Reference** |
| --- | --- | --- | --- | --- | --- | --- | --- |
| ***Bathydraco marri*** | 300-1250 | D | *Lepidapedon garrardi* | Weddell Sea | 7.1 | 1.0 (1) | [1,2] |
|  |  | D | *Steringophorus arntzi* | Weddell Sea | 14.3 | (1-2) | [2,3] |
|  |  | C | Tetraphyllidea indet. | Weddell Sea | 29.0 | 1.0 (1) | [4] |
| ***Gerlachea australis*** | 200-670 | D | *Neolebouria antarctica* | Scotia Sea | 20.0 | 1.0 (1) | this study |
|  |  | D | Digenea indet. | Scotia Sea | 20.0 | 1.0 (1) | this study |
|  |  | C | Tetraphyllidea indet. | Weddell Sea | 25.0 | 1.0 (1) | [4] |
|  |  | N | *Contracaecum osculatum* s.l. | Scotia Sea | 40.0 | 1.5 (1-2) | this study; [5] |
|  |  | N | *Contracaecum radiatum* | Scotia Sea | 20.0 | 1.0 (1) | this study; [5] |
|  |  | N | *Contracaecum* sp. | Scotia Sea | 40.0 | 4.0 (2) | this study |
|  |  | N | Nematoda indet. | Scotia Sea | 40.0 | 1.0 (1) | this study |
| ***Racovitzia glacialis*** | 219-610 | D | *Otodistomum cestoides* | Weddell Sea | 20.0 | (1-2) | [2,3] |
|  |  | D | *Caudotestis glacialis* | South Shetland Islands;  Weddell Sea;  Adelie Land | 59.1 | (1-2) | [2,3,6,7] |
|  |  | C | Tetraphyllidae indet. | Weddell Sea | 73.0 | (1-46) | [4] |
|  |  | N | *Ascarophis nototheniae* | Weddell Sea | 13.6; 16.7 | (1-5); 2.0 (2) | this study; [4] |
|  |  | N | *Anisakis simplex* s.s. | Scotia Sea | 16.7 | 4 (4) | this study |
|  |  | N | *Contracaecum osculatum* s.l. | Weddell Sea | 0; 33.3 | 0; 2.0 (1-3) | this study; [5] |
|  |  | N | *Contracaecum radiatum* | Scotia Sea | 33.3 | 1,5 (1-2) | this study |
|  |  | N | *Contracaecum* sp. | Scotia Sea | 50.0 | 14.0 (2-24) | this study |
|  |  | N | *Pseudoterranova decipiens* E | Scotia Sea | 33.3 | 4.0 (1-7) | this study |
|  |  | N | Nematoda indet. | Scotia Sea | 50.0 | 2.3 (1-3) | this study |
|  |  | A | *Corynosoma bullosum* | Weddell Sea | 9.1 |  | [8] |
|  |  | A | *Corynosoma pseudohamanni* | Weddell Sea | 4.5 |  | [8] |
|  |  | A | *Metacanthocephalus dalmori* | Weddell Sea | 9.1 |  | [8] |
| ***Gymnodraco acuticeps*** | 0-550 | D | *Neolebouria antarctica* | Ross Sea; Scotia Sea | 25.0; 11.1 | 1.0 (1) | this study |
|  |  | D | *Neolebouria terranovaensis* | Ross Sea |  |  | [9] |
|  |  | D | *Genolinea bowersi* | Ross Sea | 50.0 | 6.5 (3-10) | [9] |
|  |  | D | *Elytrophalloides oatesi* | Ross Sea | 25.0 | 3 (3) | [9] |
|  |  | D | Digenea indet. | Scotia Sea | 11.1 | 1.0 (1) | this study |
|  |  | C | Cestoda indet. | Ross Sea;  South Shetland Islands |  |  | [9] |
|  |  | C | Tetraphyllidea indet. | Scotia Sea | 11.1 | 1.0 (1) | this study |
|  |  | N | *Ascarophis nototheniae* | Ross Sea | 25.0 | 6.0 (6) | [9] |
|  |  | N | *Contracaecum osculatum* s.l. | Scotia Sea | 88.9 | 12.9 (1-56) | this study |
|  |  | N | *Pseudoterranova decipiens* E | Scotia Sea | 11.1 | 4.0 (4) | this study |
|  |  | N | Nematoda indet. | Scotia Sea | 33.3 | 2,7 (1-4) | this study |
|  |  | A | *Corynosoma bullosum* | Scotia Sea | 11.1 | 1 (1) | this study |
|  |  | A | *Corynosoma pseudohamanni* | Ross Sea | 50.0 | 36.5 (1-72) | [9] |
|  |  | A | Acanthocephala indet. | Scotia Sea | 11.1 | 1 (1) | this study |
| ***Parachaenichthys georgianus*** | 5-270 | D | *Gonocerca phycidis* | South Shetland Islands |  |  | [6] |
|  |  | D | *Elytrophalloides oatesi* | South Georgia Island |  |  | [6] |
|  |  | D | *Genolinea bowersi* | South Georgia Island |  |  | [6] |
|  |  | D | *Lecithaster macrocotyle* | South Georgia Island |  |  | [6] |
|  |  | D | *Lecithaster micropsi* | Shag Rock |  |  | [6] |
|  |  | D | *Neolebouria antarctica* | South Shetland Islands,  South Georgia Island |  |  | [6] |
|  |  | N | *Ascarophis nototheniae* |  |  |  | [10] |
|  |  | N | *Dichelyne fraseri* |  |  |  | [10] |
|  |  | A | *Heterosentis heteracanthus* |  |  |  | [11*] |
|  |  | A | *Corynosoma arctocephali* | South Georgia Island |  |  | [11*] |
|  |  | A | *Corynosoma bullosum* | South Georgia Island |  |  | [11*] |
|  |  | A | *Corynosoma shackletoni* | South Georgia Island |  |  | [11*] |
|  |  | A | *Andracantha baylisi* | South Georgia Island |  |  | [11*] |
|  |  | A | *Metacanthocephalus dalmori* | South Gerogia Island |  |  | [11*] |
|  |  | H | *Nototheniobdella sawyeri* | Weddel Sea |  |  | [12] |
|  |  | H | *Trulliobdella capitis* | South Georgia Island |  |  | [12,13] |
|  |  | H | *Trulliobdella bacilliformis* | Soutje Georgia Island |  |  | [12] |
| ***Parachaenichthys charcoti*** | 5-400 | D | *Genolinea bowersi* | South Shetland Islands | 26.7 | (2-4) | [6,14*,15*] |
|  |  | D | *Elytrophalloides oatesi* | South Shetland Islands |  |  | [6,14*,15*] |
|  |  | D | *Glomericirrus macrouri* | South Shetland Islands | 6.7 |  | [6,15*] |
|  |  | D | *Gonocerca phycidis* | Scotia Sea | 2.1 | 2.0 (1-2) | this study |
|  |  | D | *Lecithaster macrocotyle* | Scotia Sea | 2.1 | 1.0 (1) | this study;  [6,15*–17] |
|  |  | D | *Lecithaster* sp. | Scotia Sea | 2.1 | 1.0 (1) | this study |
|  |  | D | *Lepidapedon garrardi* | South Shetland Islands |  |  | [6,15*] |
|  |  | D | *Lepocreadium trullaforme* |  |  |  | [14*] |
|  |  | D | *Macvicaria georgiana* | South Shetland Islands |  |  | [3,15*] |
|  |  | D | *Neoleburia antarctica* | South Shetland Islands |  |  | [6,14*,15*,18*] |
|  |  | C | Tetraphyllidea indet. | Scotia Sea | 27.6 | 3.0 (1-9) | this study |
|  |  | C | Pseudophyllidea sp. | South Shetland Islands |  |  | [17] |
|  |  | C | Scolex pleuronectis | South Shetland Islands |  |  | [14*] |
|  |  | N | *Ascarophis nototheniae* | Scotia Sea | 2.1 | 1.0 (1) | this study, [17] |
|  |  | N | *Contracaecum osculatum* s.l. | Scotia Sea | 25.5 | 3,8 (1-15) | this study, [17] |
|  |  | N | *Contracaecum radiatum* | Scotia Sea | 2.1 | 1.0 (1) | this study, [17] |
|  |  | N | *Contracaecum* sp. | Scotia Sea, South Shetland  Islands,  Admiralty Bay,  Barnsfield Strait | 2.1 | 1.0 (1) | this study; [14*,15*,17] |
|  |  | N | *Pseudoterranova decipiens* E | Scotia Sea | 57.5 | 3,5 (1-14) | this study, [17] |
|  |  | N | *Pseudoterranova decipiens* s.l. | South Shetland Islands,  Admiralty Bay |  |  | [15*,19] |
|  |  | N | Nematoda indet. | Scotia Sea | 34.0 | 1,5 (1-4) | this study |
|  |  | A | *Aspersentis megarhynchus* | South Shetland Islands,  Admiralty Bay |  |  | [14*,15*,17,20*,21] |
|  |  | A | *Corynosoma arctocephali* | South Shetland Islands,  Admiralty Bay |  |  | [15*,21] |
|  |  | A | *Corynosoma australe* | Scotia Sea | 2.1 | 1.0 (1) | this study |
|  |  | A | *Corynosoma bullosum* | Scotia Sea | 8.5 | 1.0 (1) | this study, [15*,17,21] |
|  |  | A | *Corynosoma hamanni* | South Shetland Islands,  Admiralty Bay |  |  | [15*,21] |
|  |  | A | *Corynosoma pseudohamanni* | South Shetland Islands |  |  | [15*,21] |
|  |  | A | *Corynosoma shackletoni* | South Shetland Islands |  |  | [21] |
|  |  | A | *Corynosoma* sp. | Scotia Sea | 2.1 | 1.0 (1) | this study; [17] |
|  |  | A | *Metacanthocephalus dalmori* | Scotia Sea, Admiralty Bay,  Bransfield Strait | 8.5 | 1.0 (1) | this study, [15*,21] |
|  |  | A | *Metacanthocephalus campbelli* | South Shetland Islands |  |  | [15*,21] |
|  |  | A | *Metacanthocephalus johnstoni* | South Shetland Islands |  |  | [15*] |
|  |  | A | *Metacanthocephalus* sp. | South Shetland Islands |  |  | [17] |
|  |  | A | *Hypoechinorhynchus magellanicus* | South Shetland Islands |  |  | [14] |
|  |  | A | Acanthocephala indet. | Scotia Sea | 8.5 | 1,5 (1-3) | this study |
|  |  | Cr | *Eubrachiella antarctica* | Scotia Sea | 2.1 | 1.0 (1) | this study |
| ***Prionodraco evansii*** | 70-550 | D | *Elytrophalloides oatesi* | Weddell Sea |  | (1-5) | [2,22*] |
|  |  | D | *Lepidapedon garrardi* | Weddell Sea | 66.7 | (1-2) | [2,23*,24] |
|  |  | C | Tetraphyllidea indet. | Weddell Sea | 100 | (5-40) | [4] |
|  |  | N | *Contracaecum* sp. | Weddell Sea |  | (1-5) | [22*] |
|  |  | A | *Corynosoma bullosum* | Weddell Sea | 33.3 |  | [8] |
|  |  | Cr | *Eubrachiella antarctica* | Weddell Sea | 20.0% | (1-2) | [22*] |
| ***Cygnodraco mawsoni*** | 112-300 | D | *Neolebouria terranovaensis* | Ross Sea | 100 | 192 (192) | [9] |
|  |  | D | *Genolinea bowersi* | Ross Sea | 100 | 1 (1) | [9] |
|  |  | D | *Elytrophalloides oatesi* | Ross Sea | 100; 100 | 24 (24); 11 (11) | [9] |
|  |  | D | Digenea indet. | Ross Sea | 100 | 61 (10-112) | [25] |
|  |  | C | Tetraphyllidea indet. | Ross Sea | 50 | 4678 | [25] |
|  |  | C | Cestoda indet. | Ross Sea | 100; 100 | 106 (106); 7 (7) | [9] |
|  |  | N | *Ascarophis nototheniae* | Ross Sea | 100 | 1 (1) | [9] |
|  |  | N | *Contracaecum osculatum* s.l. | Ross Sea | 100 | 160.5(114-207) | [25] |
|  |  | N | *Contracaecum* sp. | Ross Sea | 100 | 1 (1) | [9] |
|  |  | N | *Pseudoterranova decipiens* s.l. | Weddell Sea |  |  | [5] |
|  |  | A | Acanthocephala indet. | Ross Sea | 100 | 28 (7-47) | [25] |
| ***Psilodraco breviceps*** | 60-345 | D | *Neoleburia antarctica* | South Georgia | 100 | 2.0 (2) | [6] |
|  |  | D | *Elytrophalloides oatesi* | South Georgia | 100 | 8.0 (3-12) | [6] |
|  |  | D | *Lecithaster macrocotyle* | South Georgia | 33.3 | 1.0 (3) | [6] |
|  |  | C | Diphyllobothrium indet. | South Gerogia |  |  | [26] |
| ***Vomeridens infuscipinnis*** | 500-813 | N | *Hysterothylacium aduncum* | Dallman bay |  |  | [27] |
| ***Acanthodraco dewitti*** | ?-255 | N | *Contracaecum* sp. | King George Island, Admiralty Bay |  | 4.0 (4) | [28] |
|  |  | N | *Pseudoterranova decipiens* s.l. | King George Island, Admiralty Bay |  | 1.0 (1) | [28] |

1. Zdzitowiecki K, Cielecka D. Digenea of fishes of the Weddell Sea. III. The Lepocreadiidae (genera *Neolepidapedon* and *Lepidapedon*), parasites of Notothenioidea. Acta Parasitol. 1997;42:84–91.

2. Zdzitowiecki K. Occurrence of Digenea in fishes of the family Bathydraconidae in the Weddell Sea and other areas of Antarctica. Acta Parasitol. 2002;47:310–13.

3. Zdzitowiecki K. Antarctic Digenea, parasites of fishes. Koenigstein: Koeltz Scientific Books; 1997.

4. Rocka A. The tetraphyllidean cercoids from teleosts occurring in the Weddell Sea (Antarctic). Acta Parasitol. 1999;44:115–18.

5. Klöser H, Plötz J, Palm H, Bartsch A, Hubold G. Adjustment of anisakid nematode life cycles to the high Antarctic food web as shown by *Contracaecum radiatum* and *C. osculatum* in the Weddell Sea. Antarct Sci. 1992;4:171–78.

6. Zdzitowiecki K. Occurrence of digeneans in open sea fishes off the South Shetland Islands and South Georgia, and a list of fish digeneans in the Antarctic. Pol Polar Res. 1991;12:55–72.

7. Zdzitowiecki K, Ozouf−Costaz C. Contribution to the knowledge of the parasitic fauna of fish off Adelie Land, Antarctica. Pol Polar Res. 2013;34:429–35.

8. Zdzitowiecki K. Acanthocephala in fish in the Weddell Sea (Antarctic). Acta Parasitol. 1996;41:199–203.

9. Laskowski Z, Rocka A, Zdzitowiecki K, Ghigliotti L, Pisano E. New data on the occurrence of internal parasitic worms in the *Gymnodraco acuticeps* and *Cygnodraco mawsoni* (Bathydraconidae) fish in the Ross Sea, Antarctica. Pol Polar Res. 2005;26:37–40.

10. Rocka A. Helminths of Antarctic fishes: Life cycle biology, specificity and geographical distribution. Acta Parasitol. 2006;51:26–35.

11. Zdzitowiecki K, others. Occurrence of acanthocephalans in fishes of the open sea off the South Shetlands and South Georgia (Antarctic). Acta Parasitol. Pol. 1990;35:131–141.

12. Utevsky AY. An identification key to Antarctic fish leeches (Hirudinea: Piscicolidae). Vestn Zool. 2005;3:135–44.

13. Brinkmann A. 2 New Antarctic Leeches. Nature. 1947;160:756–56.

14. Szidat L, Graefe G. Estudios sobre la fauna de parasitos de peces antarticos. Armada Argentina, Servicio de Hidrografía Naval de la Secretaria de la Rep. Argentina; 1967.

15. Zdzitowiecki K. Occurrence of endoparasitic worms in a fish, *Parachaenichthys charcoti* (Bathydraconidae), off the South Shetland Islands (Antarctica). Acta Parasitol. 2001;46:18–23.

16. Zdzitowiecki K. Antarctic representatives of the genus *Lecithaster* Luhe, 1901 (Digenea, Hemiuridae), with the description of a new species. Acta Parasitol. 1992;37:57–63.

17. Palm HW, Klimpel S, Walter T. Demersal fish parasite fauna around the South Shetland Islands: high species richness and low host specificity in deep Antarctic waters. Polar Biol. 2007;30:1513–22.

18. Zdzitowiecki K. A contribution to the morphology of the Antarctic fish lepocreadiid digeneas, with a description of a new genus. Acta Parasitol. 1993;38:109–112.

19. Palm HW. Ecology of *Pseudoterranova decipiens* (Krabbe, 1878)(Nematoda: Anisakidae) from Antarctic waters. Parasitol Res. 1999;85:638–646.

20. Zdzitowiecki K, Rokosz B. Prevalence of acanthocephalans in fishes of South Shetlands (Antarctic). II. *Aspersentis austrinus* Van Cleave, 1929 and remarks on the validity of Heteracanthocephalus hureaui Dollfus, 1965. Acta Parasitol Pol. 1986;30:161–71.

21. Zdzitowiecki K. Antarctic acanthocephala. Koeltz Scientific Books; 1991.

22. Kock KH, Schneppenheim R, Siegel V. A contribution to the fish fauna of the Weddell Sea. Arch Fisch. 1984;34:103–120.

23. Prudhoe S, Bray RA. Digenetic trematodes from fishes. BANZ Antarc Res Exp Rep Ser B. 1973;8:195–225.

24. Zdzitowiecki K, Cielecka D. Digenea of fishes of the Weddell Sea. I. Parasites of *Macrourus whitsoni* (Gadiformes, Macrouridae). Acta Parasitol. 1997;42:23–30.

25. Santoro M, Mattiucci S, Work T, Cimmaruta R, Nardi V, Cipriani P, Bellisario B, Nascetti G. Parasitic infection by larval helminths in Antarctic fishes: pathological changes and impact on the host body condition index. Dis Aquat Org. 2013;105:139–148.

26. Wojciechowska A. The tetraphyllidean and tetrabothriid cercoids from Antarctic bony fishes. II. Occurrence of cercoids in various fish species. Acta Parasitol. 1993;38.

27. Oguz MC, Heckmann RA, Cheng CC, El-Naggar A, Tepe Y. Ecto and endoparasites of some fishes from the Antarctic region. Sci. Parasitol. 2012;13:119–128.

28. Rokicki J, Rodjuk G, Zdzitowiecki K, Laskowski Z, others. Larval ascaridoid nematodes (Anisakidae) in fish from the South Shetland Islands (Southern Ocean). Pol. Polar Res. 2009;30:49–58.
